# TRIM28 Is a Novel Regulator of CD133 Expression Associated with Cancer Stem Cell Phenotype

**DOI:** 10.3390/ijms23179874

**Published:** 2022-08-30

**Authors:** Yan S. Kim, Daria M. Potashnikova, Alisa M. Gisina, Irina V. Kholodenko, Arthur T. Kopylov, Olga V. Tikhonova, Leonid K. Kurbatov, Aleena A. Saidova, Anna V. Tvorogova, Roman V. Kholodenko, Pavel V. Belousov, Ivan A. Vorobjev, Victor G. Zgoda, Konstantin N. Yarygin, Alexey Yu. Lupatov

**Affiliations:** 1 Laboratory of Cell Biology, V.N. Orekhovich Institute of Biomedical Chemistry, 119121 Moscow, Russia; 2 Cell Biology and Histology Department, School of Biology, M.V. Lomonosov Moscow State University, 119234 Moscow, Russia; 3 Laboratory of Systems Biology, V.N. Orekhovich Institute of Biomedical Chemistry, 119121 Moscow, Russia; 4 Transcriptome Analysis Group, Analytical Branch Department, V.N. Orekhovich Institute of Biomedical Chemistry, 119121 Moscow, Russia; 5 Department of Transcription Factors, V.A. Engelhardt Institute of Molecular Biology, Russian Academy of Sciences, 119991 Moscow, Russia; 6 Laboratory of Cell Motility, A.N. Belozersky Research Institute of Physico-Chemical Biology, M.V. Lomonosov Moscow State University, 119992 Moscow, Russia; 7 Laboratory of Molecular Immunology, M.M. Shemyakin–Yu.A. Ovchinnikov Institute of Bioorganic Chemistry of the Russian Academy of Sciences, 117997 Moscow, Russia; 8 Endocrinology Research Centre, 117292 Moscow, Russia; 9 Center for Precision Genome Editing and Genetic Technologies for Biomedicine, V.A. Engelhardt Institute of Molecular Biology, Russian Academy of Sciences, 119991 Moscow, Russia; 10 Department of Biology, School of Sciences and Humanities, Nazarbayev University, Nur-Sultan 010000, Kazakhstan; 11 Laboratory of Biophotonics and Imaging, National Laboratory Astana, Nazarbayev University, Nur-Sultan 010000, Kazakhstan

**Keywords:** cancer stem cells, CD133, cell signaling, TRIM28, stemness markers

## Abstract

CD133 is an extensively studied marker of the most malignant tumor cell population, designated as cancer stem cells (CSCs). However, the function of this glycoprotein and its involvement in cell regulatory cascades are still poorly understood. Here we show a positive correlation between the level of CD133 plasma membrane expression and the proliferative activity of cells of the Caco-2, HT-29, and HUH7 cancer cell lines. Despite a substantial difference in the proliferative activities of cell populations with different levels of CD133 expression, transcriptomic and proteomic profiling revealed only minor distinctions between them. Nonetheless, a further in silico assessment of the differentially expressed transcripts and proteins revealed 16 proteins that could be involved in the regulation of CD133 expression; these were assigned ranks reflecting the apparent extent of their involvement. Among them, the TRIM28 transcription factor had the highest rank. The prominent role of TRIM28 in CD133 expression modulation was confirmed experimentally in the Caco2 cell line clones: the knockout, though not the knockdown, of the *TRIM28* gene downregulated CD133. These results for the first time highlight an important role of the TRIM28 transcription factor in the regulation of CD133-associated cancer cell heterogeneity.

## 1. Introduction

Intratumor cell heterogeneity is one of the most intriguing research fields, due to its impact on tumor characteristics, including growth rate, invasiveness, and metastatic potential. Cancer stem cells (CSCs) constitute a subpopulation of tumor cells with the highest malignancy and their abundance correlates with a poor prognosis and tumor recurrence rate [1,2]. Prominin 1 (PROM1, CD133) is a widely used CSC marker suitable for its isolation from various tumors, including colon, lung, pancreas, and gastric cancers, as well as gliomas and many others [3,4]. Its role in the formation of the aggressive cell phenotype is not yet clear. Importantly, CD133 expression was detected in some normal cells of various human and mouse tissues, predominantly stem cells. Initially it was discovered as a marker of hematopoietic stem cells (SCs) [5], and later as a marker of stem and progenitor cells in the brain, prostate, kidney, liver, and other organs [6,7,8,9]. A positive correlation between CD133 expression in different cancers and a poor prognosis has been reported [10,11,12,13], suggesting that in-depth CD133 research can provide a basis for the development of novel cancer therapies.

It was shown that CD133 is preferably localized at plasma membrane protrusions [14] and plays an important role in the membrane’s structural arrangement [15]. CD133 is known to maintain some highly specialized membrane structures. For instance, photoreceptor degeneration following a loss of vision was observed in Prom1^−/−^ mice [16]. CD133 is associated with specific lipid rafts that have some implication in stem cell signal transduction. CD133 containing membrane microdomains may play a role in the determination of hematopoietic SCs’ fate and the course of their differentiation involves the release of CD133-containing vesicles [17]. Similar processes take place during (neuro)epithelial cell differentiation [18]. CD133-containing microparticles were found in human body fluids, suggesting their involvement in the development, regeneration, and functioning of various tissues [19].

Some of CD133’s molecular partners and related signaling pathways were disclosed. Histone deacetylase 6 (HDAC6) was shown to directly interact with CD133 and β-catenin, forming a ternary complex that prevents β-catenin acetylation and further degradation [20]. Stabilized β-catenin enters the nucleus and activates its molecular targets, resulting in the acceleration of tumor cell line growth [21]. Moreover, the phosphorylation of CD133 tyrosine-828 residue leads to the activation of the PI3K/protein kinase B (Akt) pathway by the direct interaction of CD133 with the phosphoinositide 3-kinase (PI3K) 85 kDa regulatory subunit (p85) [22]. The knockdown of CD133 inhibits the activity of the PI3K/Akt pathway and increases the survival of tumor-bearing, immunodeficient mice. The CD133 cytoplasmic tail region (aa 845–857) is involved in the interaction with Src kinase, leading to the activation of the latter [23]. Activated Src phosphorylates its substrate FAK (focal adhesion kinase) and promotes the migration of SW460 cells. In addition, there is some evidence of extracellular and intracellular stimuli involved in the regulation of CD133. Regulating stimuli encompass hypoxia [24], mitochondrial dysfunction [25], and many different molecules, including transcription factors [26], cytokines [27], and miRNA [28,29]. Despite all these findings, the function of CD133 has not been fully elucidated.

Here we present the results of the CD133 molecular regulation research carried out on Caco2, HT-29, and HUH7 cell lines. We studied the cell proliferation to confirm that CD133 has a functional significance in the used cell models. To assess the mechanisms of CD133 expression control, we applied an integrative approach, including whole transcriptome and panoramic proteome analyses, an in silico search of the CD133 key molecular regulators, and a verification of the in silico assessment with CRISPR/CAS9 gene editing.

## 2. Results

### 2.1. The Majority of Analyzed Cancer Cell Lines Do Not Contain CD133-Positive Cells

**Figure 1:**
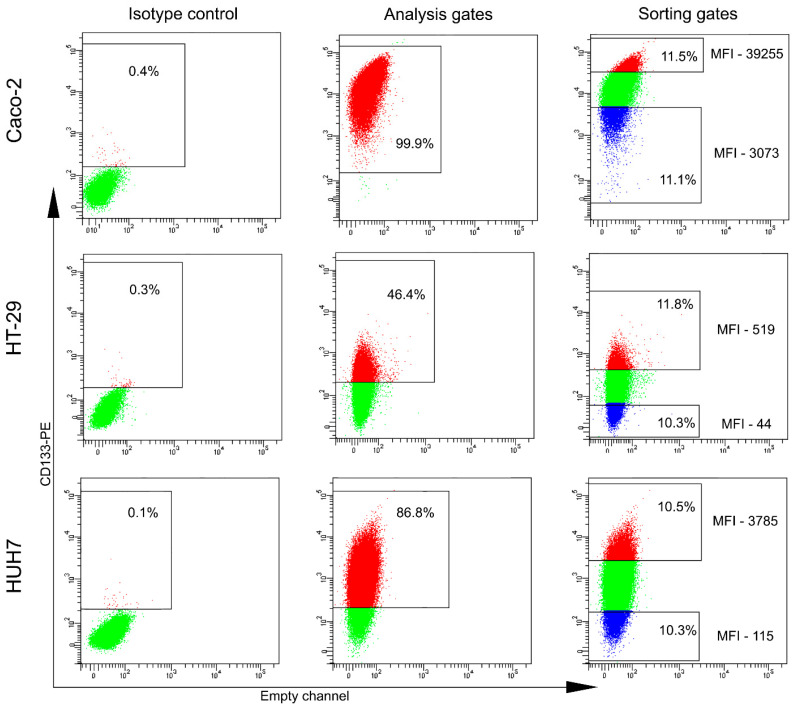
FACS analysis of CD133 expression in cancer cell lines and gating for CD133^+/high^ and CD133^−/low^ cells isolation. Left column panels: isotype controls. Middle column panels: CD133 staining of Caco2, HT-29, and HUH7 cells. FACS analysis gates were set up in accordance with the isotype controls. Right column panel: sorting gates of CD133^+/high^ (red dots) and CD133^−/low^ (blue dots) populations were set up to obtain no less than 10-fold difference between their median fluorescence intensities (MFI).

A number of tumor cell lines of different tissue origin were tested for the presence of CD133-positive cells (Table 1) and only four out of twenty-one cell lines were expressing CD133 (Figure 1 and Supplementary Figure S1). The percentages of CD133-positive cells in these lines were as follows: Caco2—99.7 ± 0.3%, HT-29—69.9 ± 21.8%, HUH7—93.4 ± 4.4%, and FTC-133—5.7 ± 4.1%. Two colon (Caco2 and HT-29) and one hepatocellular (HUH7) carcinoma cell lines comprising predominant populations of CD133-positive cells were chosen for further analysis (Table 1). In the case of Caco2 having a small CD133-negative cell population, the sorted CD133^−/low^ population encompassed both cells with negative and with low levels of CD133 expression.

### 2.2. CD133^+/high^ Cells Have Higher Proliferative Capacity Than CD133^−/low^ and CD133^ref^ Cells

**Figure 2:**
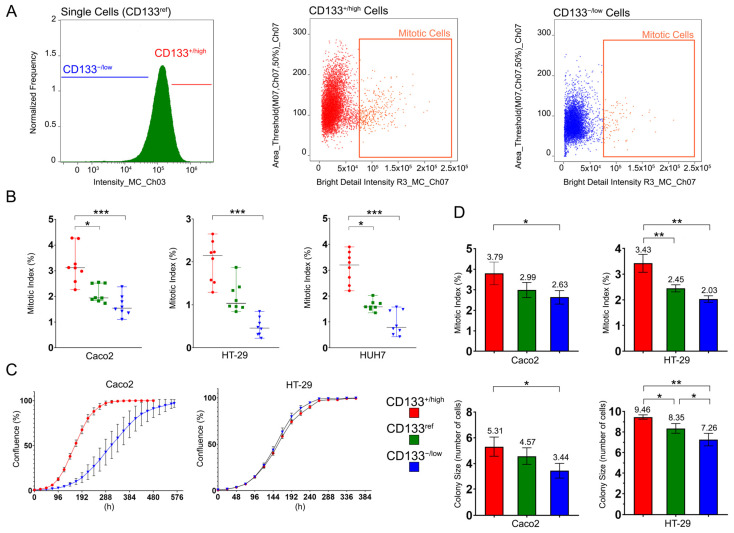
Proliferative activity of cells with different CD133 expression levels. (**A**) Adapted «ImageStream» algorithm of mitotic index (MI) counting in populations with different level of CD133 expression. CD133^+/high^ (red dots) and CD133^−/low^ (blue dots) populations are defined as cells gated at the upper and lower 10% of fluorescence intensities. CD133^ref^ denotes the original population encompassing CD133^+/high^ and CD133^−/low^ cells. (**B**) MI of populations with different CD133 expression level (*n* = 8 for each cell line; median and range). CD133^+/high^—red circles, CD133^−/low^—blue triangles and CD133^ref^—green squares. (**C**) Growth curves of populations with different CD133 expression levels (Caco2 *n* = 6, HT-29 *n* = 5; mean ± SD). (**D**) MI (top panel row) and the number of cells (bottom panel row) in three-day colonies formed by CD133^+/high^ (red columns), CD133^−/low^ (blue columns), and CD133^ref^ (green columns) populations. * *p* ≤ 0.05; ** *p* ≤ 0.01; *** *p* ≤ 0.001.

We evaluated the proliferative activity in CD133^+/high^, CD133^−/low^, and CD133^ref^ cell populations by the analysis of their mitotic index (MI) using imaging flow cytometry. MI determination was carried out in CD133^+/high^ and CD133^−/low^ populations defined as cells gated at the upper and lower 10% of fluorescence intensities on the CD133-expression distribution histogram (Figure 2A). The original population combining the CD133^+/high^ and the CD133^−/low^ cells was named CD133^ref^. A Kruskal–Wallis test showed that there was a statistically significant difference in MI among the three cell populations in all three cell lines (Caco2: H = 15.92, 2 d.f., *p* = 0.0003; HT-29: H = 18.67, 2 d.f., *p* < 0.0001; HUH7: H = 19.01, 2 d.f., *p* < 0.0001). Dunn’s multiple comparisons test was applied post hoc to reveal differing groups. All three cell lines of the CD133^+/high^ populations had significantly higher MI than the CD133^−/low^ populations: the fold changes in MI medians were 2.0, 4.7, and 4.0 for Caco2, HT-29, and HUH7, respectively (Figure 2B). Moreover, the CD133^+/high^ populations of the Caco2 and HUH7 cell lines had significantly higher MI than the CD133^ref^ populations (the fold changes in MI medians were 1.6 and 2.0, respectively).

To additionally verify the relation between CD133-expression and cell proliferation activity, we determined the time necessary to form a full confluence monolayer for two cell lines—Caco2 and HT-29 (Figure 2C). The cell culture originating from the CD133^+/high^ population of Caco2 achieved full confluence much faster compared to the culture originating from the CD133^−/low^ cell population—12 and 23.5 days, respectively. Accordingly, the doubling time of the first culture was close to two times shorter than that of the last one. HT-29 cell populations with different levels of CD133, however, did not show differences in growth rates: both cultures reached full confluence in 11 days (which is equal to the doubling times for both cell cultures).

Further, we counted MI in colonies derived from CD133^+/high^, CD133^−/low^ and CD133^ref^ cells in two cell lines—Caco2 and HT-29 (Figure 2D). Since peripheral localization of most mitotic figures in large colonies could interfere with accurate quantification, MI was evaluated in three-day colonies normally consisting of no more than 10 cells. For the Caco2 and HT-29 cell lines, we calculated MI in cumulatively 3000 and 3300 colonies, respectively. In parallel, we determined the total number of cells per colony.

A one-way ANOVA test showed that there was a statistically significant difference in MI (Caco2: F(2,6) = 5.854, *p =* 0.039; HT-29: F(2,6) = 29.59, *p =* 0.0008) and colony size (Caco2: F(2,6) = 6.171, *p =* 0.035; HT-29: F(2,6) = 17.15, *p =* 0.0033) among the three cell populations in both cell lines. A Newman–Keuls multiple comparison test was applied post hoc to reveal which groups were statistically different. In both cultures, the CD133^+/high^ populations had significantly higher MIs and colony sizes than the CD133^−/low^ populations: the fold changes in the MI means were 1.4 and 1.7, and the fold changes in the colony size means were 1.5 and 1.3 for Caco2 and HT-29, respectively (Figure 2D). Moreover, the CD133^+/high^ population of HT-29 has a significantly higher MI and colony size than the CD133^ref^ population (the fold change in the means for MI—1.4; in the colony size—1.1). Moreover, the colony size in the CD133^ref^ group was higher than in the CD133^−/low^ group (the fold change in means—1.15). Thus, Caco2 and HT-29 cells with a higher expression of CD133 constitute populations with higher proliferative potential compared to cells with lower CD133 expression.

Since CD133 expression in Caco2, HT-29, and HUH7 cell lines correlates with functional activity, these cell lines may represent a suitable in vitro model for studies of regulatory pathways involving CD133.

### 2.3. Transcripts and Proteins Differentially Expressed in the CD133^+/high^ and CD133^−/low^ Cell Populations

To compare the expression profiles of the CD133^+/high^ and CD133^−/low^ cell populations isolated from the Caco2, HT-29, and HUH7 cell lines, we performed whole transcriptome and shotgun proteome analyses. The number of up- and down-regulated transcripts (with at least a two-fold difference) in the CD133^+/high^ vs. CD133^−/low^ cell populations was 7 and 3 for HT-29, 14 and 57 for Caco2, and 53 and 13 for HUH7 (Supplementary Table S1). CD133 was the only transcript up-regulated in the CD133^+/high^ population in all three cell lines.

In addition to the transcriptomic assay, we performed a comparative proteome analysis of CD133^+/high^ and CD133^−/low^ cells isolated from the same cultures. The total numbers of unique proteins identified in the three biological repeats were 2541 for Caco2, 2007 for HT-29, and 1565 for HUH7 cells. The numbers of up- and down-regulated proteins (with at least a two-fold difference) in CD133^+/high^ vs. CD133^−/low^ populations were 24 and 11 for HT-29; 17 and 22 for Caco2; and 62 and 13 for HUH7. No protein differentially expressed in CD133^+/high^ and CD133^−/low^ cells was shared by all three cell lines. Notably, one of the common markers of normal and cancer stem cells, ALDH1A1, was up-regulated in the CD133^+/high^ population of HT-29 and HUH7 cell, with fold changes of 2.9 and 1.8, respectively. Despite the distinction in differentially expressed transcripts and the proteins revealed in the three cell lines, they still could be involved in the same regulatory mechanism. To test this hypothesis, we performed an in silico analysis of CD133-associated molecular regulators.

### 2.4. In Silico Analysis of CD133-Associated Key Molecular Regulators

**Figure 3:**
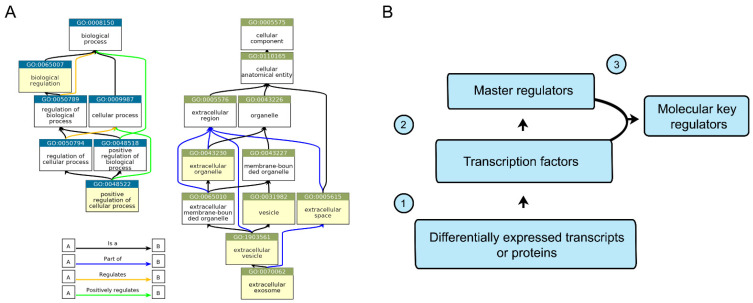
Gene Ontology classification of differentially expressed genes and the algorithm of in silico analysis of CD133 molecular key regulators. (**A**) Terms of GO classification shared by all lists of differentially expressed transcripts and proteins are highlighted in yellow. The charts were created by QuickGO browser (ebi.ac.uk/QuickGO). (**B**) Algorithm of the in silico analysis was performed by GeneXplain platform. The lists of transcriptional factors (TFs) were generated based on the lists of differentially expressed transcripts or proteins (1). The lists of master regulators (MRs) were based on the TFs lists (2). The list of key regulators was generated based on the intersection between TFs and MRs lists (3).

We formed six lists of differentially expressed genes based on the transcriptomic and proteomic analysis for each of the three studied cell lines. Each list included all differentially expressed transcripts and proteins in the CD133^+/high^ and CD133^−/low^ populations. The application of the «Gene ontology» (GO) analysis followed by the TreeMap functional classification revealed two terms shared by all gene lists in the «biological process» GO type and five terms shared in the «cellular component» GO type (Figure 3A).

Further, we analyzed the lists of differentially expressed transcripts and proteins by the GeneXplain platform in order to predict the regulator molecules controlling CD133. To predict the key CD133-associated molecular regulators (KRs, transcription factors that may act as master regulators), we employed two databases. TRANSFAC^®^ was utilized to generate the lists of transcription factors (TFs) based on the lists of differentially expressed transcripts and proteins controlled by them. TRANSPATH^®^ was employed to generate the lists of molecules (master regulators, MRs) that are on top of the hierarchy of regulatory pathways (Figure 3B). In total, six lists of TFs and six lists of MRs were generated. The final list of KRs was set up by the intersection of the TF and MR lists.

The numbers of TFs and MRs for differentially expressed transcript lists were 99 and 21 for Caco2; 50 and 64 for HT-29; and 87 and 74 for the HUH7 cell line. The respective numbers of TFs and MRs for differentially expressed protein lists were 52 and 17 for Caco2; 83 and 29 for HT-29; and 52 and 39 for the HUH7 cell line. TFs and MRs present only in one list were discarded (Supplementary Table S2). As a result, we obtained 117 TFs and 57 MRs shared by two to six individual lists. The list of KRs included 16 different entries (Table 2). To evaluate the relevance of the obtained TFs, MRs, and KRs, we applied a rank system by assigning one point for the presence of a certain TF or MR in one of the six lists. The ranks of KRs were calculated by summing up the ranks of corresponding TFs and MRs.

The highest ranked TF was GCM2, which had six points. TRIM28, RELA, IKZF1, and KLF6 gained five points and 112 other transcription factors had two to four points. TRIM28 (also known as KAP1 or TIF1-β) had the highest rank in the MR lists with five points, while MYB had four points, and 55 other MRs had two to three points. The following KRs were awarded the highest ranks: TRIM28 (ten points), RELA (eight points), and MYB (eight points). The other KRs had four to six points.

Thus, several proteins that could be involved in the upstream regulation of CD133 were predicted. None of these potential key regulators showed differential expression between CD133^+/high^ and CD133^−/low^ populations in all three cell lines, even at the transcriptome level (Supplementary Table S1). We chose KR with the maximal rank, TRIM28, and verified its involvement in CD133 regulation by two in vitro approaches—gene knockdown by the shRNA lentivirus transduction, and full gene knockout using the CRISPR/CAS9 gene editing tool.

### 2.5. Full TRIM28 Knockout, But Not Its Knockdown Downregulates CD133 Expression

We generated a modified Caco2 cell line with TRIM28 knockdown by an shRNA lentivirus transduction. Despite the clear downregulation of TRIM28 (Supplementary Figure S2A), the level of CD133 expression compared to the shRNA negative control was not affected (Supplementary Figure S2B–D; Kruskal–Wallis H = 22.06, 2 d.f., *p =* 0.0907 with post hoc Dunn’s test). On the other hand, Caco2 cells treated with the control shRNA or the anti-TRIM28 shRNA both displayed CD133 downregulation compared to the wild-type cells (Supplementary Figure S2B–D; Kruskal–Wallis H = 22.06, 2 d.f., *p <* 0.0001 and *p* < 0.0349, respectively, with post hoc Dunn’s test), probably due to the toxic effect of puromycin.

Another approach to verify our in silico analysis results was based on the full TRIM28 depletion by CRISPR/CAS9-mediated knockout. We generated 11 fully *TRIM28* knockout clones («KO» group); 19 clones with attempted but failed *TRIM28* knockout as defined by a western blot («failed KO» group); 10 clones with attempted knockout using gRNA targeted to non-mammalian *tagRFP* gene («reference KO» group, negative control); and 11 clones generated without a knockout procedure by cloning Caco2 cells («untreated clones»). Wild-type Caco2 culture was used as an additional control in several biological repeats (*n* = 14; «wild type» group). TRIM28 expression was tested by a western blot (Supplementary Figure S3). The level of CD133 expression was analyzed by FACS.

**Figure 4:**
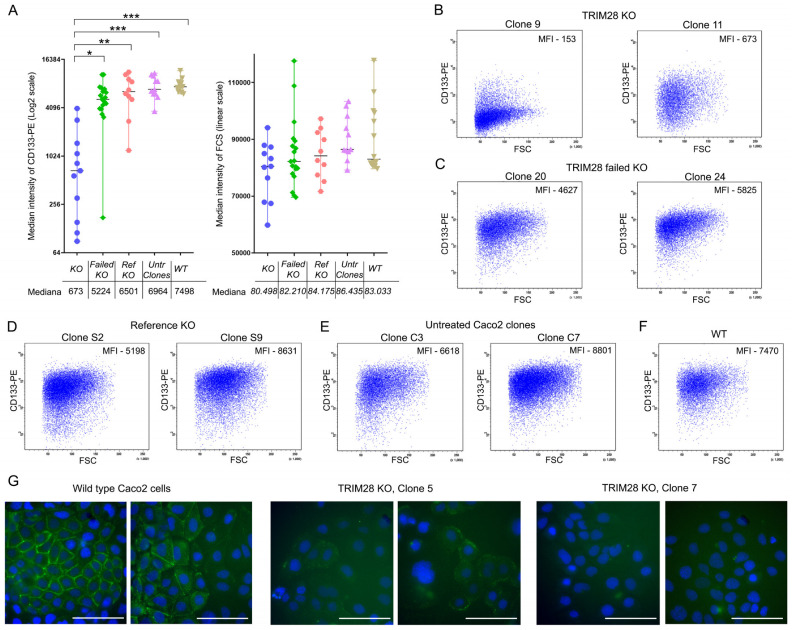
Downregulation of CD133 by full depletion of TRIM28 in Caco2 clones. (**A**) Left graph: CD133-PE median fluorescence intensity (MFI) of cells in full *TRIM28* knockout and negative control groups (Log2 scale). Right graph: forward scatters (FSC) of cells in full *TRIM28* knockout and negative control groups (linear scale). «KO» —clones with full knockout of *TRIM28* (*n* = 11, blue circles); «Failed KO»—clones with attempted but failed *TRIM28* knockout (*n* = 19, green diamonds); «Ref KO»—clones with attempted knockout using gRNA targeted to *tagRFP* non-mammalian gene (*n* = 10, pink hexagons); «Untr Clones»—«untreated» clones generated without the knockout procedure (*n* = 11, violet triangles); «WT»—wild-type cells analyzed in 14 independent experiments (yellow triangles). (**B**–**F**) Representative FACS plots of CD133 expression in analyzed groups. (**G**) CD133 immunocytochemical staining (green color) in live Caco2 cells with or without *TRIM28* knockout. Scale bar—100 µm. * *p* ≤ 0.05; ** *p* ≤ 0.01; *** *p* ≤ 0.001.

The Kruskal–Wallis test revealed a statistically significant difference in CD133 expression among five groups (H = 31.5, 4 d.f., *p* < 0.0001); the post hoc Dunn’s test showed that KO cells had significantly lower CD133 expression compared to each of the other groups (Figure 4). Fluorescence intensity was higher in the failed KO, reference KO, untreated clones, and wild-type groups compared to the KO group (median MFI fold changes were 7.8, 9.7, 10.3, and 11.1, respectively). No significant differences in the CD133 expression were found among the first four groups, confirming the specificity of the *TRIM28* knockout effect (*p* > 0.05 for each compared pair by post hoc Dunn’s test).

Since cell size can affect fluorescence intensity, we evaluated forward scatter that normally correlates with cell size. No significant differences were found by the Kruskal–Wallis test among all five groups (H = 7.963, 4 d.f., *p* = 0.092).

In addition, we verified the CD133 downregulation Caco2 *TRIM28* KO clones by fluorescence microscopy (Figure 4G). In contrast to the wild-type Caco2 cells showing bright membrane fluorescence, the KO clones demonstrated no or only subtle fluorescence.

Thus, we showed that full depletion of TRIM28 downregulates CD133 expression and TRIM28 can be considered as a novel regulator of the CD133-associated heterogeneity of cancer cells.

## 3. Discussion

The interrelation between CD phenotype and the functional properties of cancer cell populations is currently the focus of extensive research. In the present study, we evaluated CD133 expression in 21 tumor cell lines (Table 1) and found major populations of CD133-positive cells in Caco2 and HT-29 (both colorectal cancer cell lines), HUH7 (hepatocellular carcinoma cell line), and a minor population in FTC-133 (thyroid cancer cell line). Most of the cell lines did not express the plasma membrane CD133 at all, including three other tested colorectal cell lines. These data appear to contradict our clinical observations showing that just one among 30 colon cancer patients’ samples lacked CD133-positive cells [30]. Like in cell lines, in individual tumor samples, the CD133-positive share varied in broad limits: from 1 to 65%. Perhaps CD133 is not a mandatory protein for the adaptation of cancer cells for growth in vitro. Accordingly, the CD133 expression by cells of some cancer cell lines could be lost during maintenance in culture.

Although the exact functional role of CD133 remains elusive, there is some evidence shedding light on it. Thus, cancer cell populations with higher levels of CD133 usually show more aggressive behavior than cancer cell populations with lower or no CD133 expression [31,32]. Increased tumorigenicity remains the main hallmark of CSCs. Therefore, the appropriate cell line model for CSC study should demonstrate the correlation between the marker of interest and malignancy-associated functional activity.

Indeed, we demonstrated a close relationship between CD133 expression and growth characteristics of Caco2, HT-29, and HUH7 cells. The adapted ImageStream algorithm allowed us to compare MI in CD133^+/high^, CD133^−/low^, and CD133^ref^ populations of these cell lines (Figure 2A and Supplementary Figure S4). Cancer cell populations with a higher level of CD133 showed a higher mitotic index than populations with a lower level of CD133 (Figure 2B).

The growth curve analysis showed a direct correlation between the CD133 expression and the growth rate for Caco2, but not for HT-29 cells. Such contradictory results could be related to the insufficient difference in actual CD133 expression in CD133-negative and CD133^low^ HT-29 cells, leading to indistinguishable differences in cell growth rates as measured by the confluence analysis (Figure 1). Further employment of a more accurate assay allowed us to affirm the direct correlation between proliferation activity and CD133 expression for both Caco2 and HT-29 cell lines (Figure 2D). The HUH7 cell line was excluded from the experiment because of its inability to grow in colonies originating from single cells. Our in vitro results are in line with the in vivo studies in immunodeficient mice revealing the high proliferative activity of the CD133-overexpressing CSC population [3].

Both transcriptome and proteome studies revealed only modest distinctions between CD133^+/high^ and CD133^−/low^ cell populations (Supplementary Table S1). We observed no correlation between mRNA and protein expression, a discrepancy common in postgenomic research [33,34,35]. Transgelin (TAGLN) down-regulated in CD133^+/high^ Caco2 cells (the fold changes for the transcriptome set were 0.44 and for proteome were 0.48) was the only differentially expressed gene shared by the transcriptome and the proteome datasets.

As revealed by the proteomic approach, the common marker of CSCs and normal SCs, ALDH1, was up-regulated in CD133^+/high^ populations of HT-29 and HUH7, though in HUH7 cells its fold change was slightly below 2 (fold change 1.8). The co-expression of CD133 and ALDH1 is associated with the CSC population in various cancers, including colon and liver carcinomas, as verified by immunostaining and a western blot, respectively [36,37].

The GO «biological process» analysis revealed two shared terms for all lists of the differentially expressed transcripts and proteins: «positive regulation of cellular process» and «biological regulation» (Figure 3A), which is too general to clarify CD133-associated features. In contrast, the GO «cellular component» analysis showed five shared terms that have specific features related to extracellular space and exosomes. This finding is in agreement with the data demonstrating that CD133 containing extracellular particles could be involved in maintaining such biological processes as tissue development and the regulation of cell proliferation and differentiation [17,38], as well as in some pathological states [39].

To reveal molecular regulators of CD133, we analyzed each list of differentially expressed genes by the GeneXplain platform (Figure 3B and Table 2). TRIM28, the KR with the highest rank, regulates a wide spectrum of intracellular processes, including transcriptional elongation [40], response to DNA damage [41], mediation of epithelial-mesenchymal transition [42], p53 inactivation [43], maintenance of SCs pluripotency [44], and others. However, there is no evidence about its participation in CD133 regulation. Meanwhile, the ability of some of the predicted KRs to affect CD133 has been already demonstrated in a number of recent publications. HIF1A (hypoxia-inducible factor 1-alpha) up-regulates CD133 under hypoxia conditions, and promotes tumor growth and tumor-initiating and metastatic activities [24,45,46]. Moreover, HIF1A can induce CD133 expression in cooperation with another predicted KR, RELA, which is also known as the p65 subunit of NF-kB [47]. Interestingly, not only CD133’s expression, but also its glycosylation status can be altered under hypoxia conditions [48]. P53 can also down-regulate CD133 expression by binding to the non-canonical p53-binding sequence in the CD133 promoter [49]. NANOG is involved in maintaining cell pluripotency in embryonic SCs and is expressed by several types of adult SCs [50]. NANOG is able to up-regulate the expression of CD133 and some other CSCs markers [51]. Despite some resemblance between molecular markers of normal SCs and CSCs, the issue of CSCs’ origin from normal SCs is still open for debate [52]. Perhaps, their similarity is a result of CSCs’ functional and phenotypic plasticity [53]. There is some evidence of CD133 co-expression with EGR1 and HMGA1 [54,55], although the role of these transcription factors in CD133 regulation is poorly understood. Thus, some of the sixteen predicted KRs were previously shown as regulators of CD133, which emphasizes the relevance of the results of our in silico analysis.

To verify the involvement of the predicted KR with the highest rank, TRIM28, in CD133 regulation, we carried out knockdown and knockout experiments with the Caco2 cell line. The Caco2 cells display a wider distribution of the CD133 expression level than the HT-29 and HUH7 cells (Figure 1), allowing the detection of even minor changes in the CD133 expression. The downregulation of TRIM28 by shRNA did not affect CD133 expression, suggesting that even a decreased amount of TRIM28 could maintain the CD133 level, as was shown for some other transcription factors that could activate transcription at very low concentrations [56,57]. The difference in the effects of large and small amounts of certain transcription factors on target genes expression was also demonstrated in vivo. For example, the decreased level of Nanog in the inner cell mass of Nanog^+/−^ mouse embryos was able to suppress Cdx2 expression, while the Nanog^−/−^ embryos were positive for Cdx2 [58]. The discrepancy between knockdown and knockout effects was reviled in a murine model comparing mice treated with Ppara siRNA and Ppara knockout animals [59]. Based on these data, we used CRISPR/CAS9 gene editing to achieve the maximum reduction in TRIM28 expression.

We analyzed CD133 expression in 11 clones with full TRIM28 depletion and in 40 clones included in the three negative control groups (Figure 4 and Supplementary Figure S3). To minimize potential bias of cloning because of parental cell line heterogeneity, we used a sufficiently high number of clones. Since CD133 downregulation and expressions close to zero were demonstrated in most of the *TRIM28* knockout clones (Figure 4), TRIM28 can be regarded as a relevant molecular regulator of CD133 expression.

TRIM28 has a role in the regulation of human iPS cells pluripotency through its RING and PHD domains [60]. It is a specific marker of glioblastoma stem-like cells and participates in their invasion [61,62]. TRIM28 knockdown in MDA-MB-231 breast cancer cells decreases the number of CSCs and their tumorigenicity in the xenograft assay [63]. Moreover, TRIM28 overexpression in MCF7 cells increases the size and number of formed mammospheres, as well as the percentage share of CSCs (CD44^+^CD24^−^ cells), whereas the depletion of TRIM28 in MCF7 and T47D cells inhibits mammosphere formation [64]. Melanoma tumors with high TRIM28 expression were depleted of infiltrating immune cells and are characteristic of patients with poor outcomes, while melanoma cell cultures with high expression of TRIM28 have an enhanced ability to form melanosphere [65]. TRIM28 expression correlates with poor prognoses in patients with glioma [66], hepatocellular carcinoma [67], ovarian cancer [68], and early-stage non-small cell lung cancer [69]. The complexity of TRIM28’s role and its clinical significance are summarized in a comprehensive review [70]. Thus, similar to CD133, the expression of TRIM28 positively correlates with the more malignant state of cancer cells and an unfavorable clinical outcome.

Our work for the first time demonstrates the role of TRIM28 as a regulator of CD133 expression. This finding provides better insight into the molecular mechanisms of CD133-associated intratumor cell heterogeneity and can contribute to the development of target therapy against CSCs.

## 4. Materials and Methods

### 4.1. Cell Culture

All cell lines (Table 1) were provided by a laboratory of cell biology (Institute of Biomedical Chemistry, Moscow) and were maintained in a DMEM/F-12-based medium (1:1), except MDA-MB-231 cultured in a RPMI-1640 medium. The media were supplemented with 10% FBS (except Caco2—20% FBS; Thermo Fisher Scientific, Waltham, MA, USA), 2 mM L-glutamine, 100 U/mL penicillin, and 100 ug/mL streptomycin. All cell lines were cultured at 37 °C in the presence of 5% CO_2_ for no longer than 10 passages after thawing. All cell lines were routinely tested for mycoplasma contamination using MycoReport (Evrogen, Moscow, Russia) and authenticated by an STR profile analysis.

### 4.2. FACS (Fluorescence-Activated Cell Sorting)

Cells were detached from plastic by accutase (Thermo Fisher Scientific, Waltham, MA, USA) at 70–90% confluence, washed, and stained with anti-CD133-PE antibodies (clone AC133, Miltenyi Biotec, Bergisch Gladbach, Germany) following the manufacturer’s protocol. Cell sorting and flow cytometry analyses were performed using FACSAria instruments, and data were analyzed using BD FACSDiva software (BD Biosciences, Franklin Lakes, NJ, USA). Cell debris and aggregates were excluded based on forward and side scatters parameters. Sorting gates for CD133^+/high^ and CD133^−/low^ cell populations were set so that the median fluorescence intensity (MFI) for the two sorted populations differed 10-fold (Figure 1). Parental cell population (CD133^ref^) was represented by living singlet cells. Dead cells were excluded by 7-AAD (7-actinomycin D; BD Biosciences, Franklin Lakes, NJ, USA) or SYTOX Blue (Thermo Fisher Scientific, Waltham, MA, USA) staining. Cells were sorted into full medium for subsequent cultivation or DMEM/F-12 (1:1) for the following proteomic and transcriptomic analyses.

### 4.3. Imaging Flow Cytometry

Imaging flow cytometry was performed using Amnis ImageStreamX Mk II Imaging Flow Cytometer (Luminex Corporation, Austin, TX, USA), and data were analyzed using IDEAS software. Cell suspensions were stained by anti-CD133-PE antibodies as mentioned above, washed, and then fixed with 4% paraformaldehyde. Nuclear staining was performed by Hoechst 33,342 (Thermo Fisher Scientific) with final concentration of 10 ug/mL. Gating strategy is described in Supplementary Figure S4. No less than 10^5^ cells were analyzed for each run.

### 4.4. Fluorescence Microscopy

Cells were grown in 35 mm Petri dishes with glass bottoms and stained with anti-CD133-VioBright FITC antibodies (clone REA753, Miltenyi Biotec, Bergisch Gladbach, Germany) and 5 ug/mL Hoechst 33,342 (Sigma-Aldrich, St. Louis, MO, USA). Live imaging was performed by inverted Zeiss Axio Observer fluorescence microscope with ×40/1 objective using Hamamatsu ORCA-Flash 4.0 V2 digital photo camera (Hamamatsu Photonics, Hamamatsu, Japan) and Zen 3.1 Blue Edition software.

### 4.5. Cell Growth Analysis

Sorted CD133^+/high^ and CD133^−/low^ cells of Caco2 and HT-29 cell lines were plated in 12-well plates (5000 and 10,000 cells per well, respectively). The Incucyte ZOOM system (Sartorius, Gottingen, Germany), was used for capture of the cell growth phase images and their further analysis. Forty-nine phase images per well were captured every 6 h using the 10× objective. Job analysis was applied for images with following parameters: segmentation adjustment—1.2 and 0.4 (Caco2 and HT-29 respectively), hole fill—8000 µm^2^ (for Caco2 only), filters—minimum 1000 and 250 µm^2^ (for Caco2 and HT-29 respectively).

### 4.6. Short-Time Clonogenic Assay and Mitotic Index (MI) Calculation

Target cell populations were plated in triplicate or quadruplicate at the density of 1000 cells per 40 mm Petri dish immediately after sorting and incubated in standard culture conditions. After 3 days, cells were rinsed with PBS and fixed for 15 min with 4% paraformaldehyde. Fixed colonies were stored in 4% paraformaldehyde at 4 °C prior to analysis. Colonies were stained with 10 µg/mL Hoechst 33,342 (Sigma-Aldrich, St. Louis, MO, USA) for 10 min and washed twice with PBS prior to the MI evaluation. Mitotic figures were counted by fluorescence microscopy. All phases from early prophase to late cytokinesis were included into the mitosis counts. One-way ANOVA and subsequent Newman–Keuls test based on three biological repeats were performed to determine the significant differences.

### 4.7. Whole Transcriptome Analysis

For total RNA preparation, 3 × 10^5^–5 × 10^5^ sorted cells were lysed in the RLT buffer (RNeasy Micro Kit; Qiagen, Hilden, Germany) following the manufacturer’s recommendations. RNA quality was analyzed by NanoDrop 1000 (Thermo Fisher Scientific, Waltham, MA, USA) and Agilent 2100 Bioanalyzer using RNA 6000 Nano assay kit (Agilent Technologies, Santa Clara, CA, USA). Only samples with RNA integrity number (RIN) above 8 were used for the following operations. Total RNA was processed using the Low RNA Input Fluorescent Linear Amplification Kit (Agilent Technologies, Santa Clara, CA, USA) according to the manufacturer’s recommendations. Briefly, cDNA synthesis was followed by the synthesis of cRNA labeled with Cy5-CTP, whereas reference RNA was labeled with Cy3-CTP. Incorporation of the fluorescent labels was monitored by NanoDrop 2000 (Thermo Fisher Scientific, Waltham, MA, USA). Whole human genome 4 * 44 K microarrays (G4112F) applying for gene expression profiling were scanned by G2565CA Microarray Scanner System and primary data were processed by the Feature Extraction v10.10.1.1 software (Agilent Technologies, Santa Clara, CA, USA). «Up and Down Identification» option of the GeneXplain platform (https://genexplain.com/genexplain-platform, accessed on 22 September 2015) was applied to reveal up- and down-regulated genes in three biological repeats with the following cut-offs: fold change at 2 and *p*-value at 0.05 with FDR correction. Microarray data are available in the ArrayExpress database (http://www.ebi.ac.uk/arrayexpress, accessed on 19 May 2022) under the accession number E-MTAB-11779.

### 4.8. Label-Free Mass-Spectrometry

Sorted cells (2 × 10^5^–4 × 10^5^ cells) were lysed in 50–100 uL of 75 mM triethylammonium bicarbonate buffer supplemented with 1% deoxycholic acid sodium salt and 5% acetonitrile, pH 8.5. Proteins were digested by sequencing grade modified trypsin (Promega). Detailed information of protein lysate processing has been given elsewhere [71]. Chromatographic separation of lysate fractions was performed on an analytical RSLC Acclaim PepMap C18 column, 150 mm length, 75 µm inner diameter, 1.8 µm particle size, 100A pore size (Thermo Fisher Scientific, Waltham, MA, USA). Collected peptide samples were analyzed using high resolution Q Exactive mass spectrometer (Thermo Fisher Scientific, Waltham, MA, USA). Protein identification was performed using the MASCOT software (www.matrixscience.com, accessed on 29 April 2015). Proteins that were presented only in one of three biological repeats were excluded. Contaminant hits were excluded utilizing two databases as described elsewhere (http://www.matrixscience.com/help/seq_db_setup_contaminants.html, accessed on 17 August 2015). Relative quantity of each protein was evaluated by the Exponentially Modified Protein Abundance Index (emPAI) [72] normalized to the geometric mean of significant peptide matches. Proteins that showed fold change higher than 2 were included in the differentially expressed proteins sets. Processed data after MASCOT peptide identification is presented in the Supplementary Table S4.

### 4.9. In Silico Analysis

Transcriptome- and proteome-derived sets of differentially expressed genes were generated by combining up- and down-regulated transcripts and proteins for each cell line individually and uploaded to the GeneXplain platform (https://genexplain.com/genexplain-platform, accessed on 27 January 2018; Wolfenbuttel, Germany). For each individual gene list, the «Full gene ontology classification» by the “biological processes”, “cellular component” and “molecular function” ontology types (minimal hits to group—2, *p*-value threshold—0.05) was applied with further building a «TreeMap on functional classification» (similarity—0.7).

Transcriptome and proteome sets were analyzed separately by the «Upstream analysis (TRANSFAC^®^ and TRANSPATH^®^)» workflow. In brief, transcription factor binding sites were identified with TRANSFAC^®^ database of the positional weight matrices (PWM) using the “vertebrate non redundant_minSUM” profile. The promoter window was selected from −1000 to +100 from the transcription start site. Obtained PWM were converted to the set of the transcription factors, which can be involved in the up- and down-regulation of gene and protein datasets. Master regulatory gene networks for up- and down-regulated transcriptomic and proteomic datasets were constructed for the transcription factors using «Regulator search» analysis using the TRANSPATH^®^ database. The following filtering cut-offs were used: maximal radius at 10, Score at 0.2, FDR at 0.05, and Z-score at 1.0.

### 4.10. Lentivirus Transduction Knockdown

shRNA against TRIM28 (NCBI Reference Sequence: NG_046945.1; Supplementary Table S3) was cloned between BamHI and EcoRI into human H1-promoter driven pGPV vector (Evrogen, Moscow, Russia) containing copGFP reporter and kanamycin resistance gene. 293T cells were co-transfected with three plasmids using calcium phosphate. In total, 20 ug of three plasmids taken in equal amounts were used: pGPV, envelope protein vector pCMV-VSV-G (plasmid #8454; Addgene) and packaging plasmid pCMV-dR8.2 dvpr (plasmid #8455; Addgene, Watertown, MA, USA). Viral supernatant was collected at 24, 48, and 72 h after transfection and concentrated using an Amicon Ultra-15 100K cutoff filter device (Millipore, Burlington, MA, USA). Viral particles concentrate was added to Caco2 cells immediately and transduced cells were selected with puromycin (6 µg/mL). Efficiency of transduction was confirmed by FACS and downregulation of TRIM28 by western blot. shRNA against firefly luciferase was used as a negative control.

### 4.11. CRISPR/CAS9-Mediated Knockout

Three gRNA sequences for CRISPR/CAS9-mediated *TRIM28* knockout (NCBI Reference Sequence: NG_046945.1; Supplementary Table S3) meeting the following criteria: (i) high efficiency score (>50%), (ii) low off-target effect (absent MM0, MM1 and MM2 off-target transcripts), (iii) location within the first exon or its close proximity were selected by the «CHOPCHOP» online tool (https://chopchop.cbu.uib.no, accessed on 10 April 2020). In case of gRNA sequences not starting with G, extra Gs were added upstream to optimize transcription from the U6 promoter. gRNA targeted to tagRFP was used as a negative control. All synthesized oligonucleotides were cloned into PX458 (pSpCas9(BB)-2A-GFP, plasmid #48138; Addgene, Watertown, MA, USA). Caco2 cells were transfected using Genjector-U (Molecta, Moscow, Russia) either with all the three TRIM28-targeted plasmids simultaneously, or with the control plasmid, and sorted using FACS in two days. GFP-positive cells were plated in a 96-well plate in complete medium at a final concentration of 1 cell per well. *TRIM28* knockout attainment was checked in all derived individual clones by western blot. gRNA sequence against tagRFP was used as a negative control.

### 4.12. SDS-PAGE and Western Blot Analysis

SDS-PAGE and western blot analysis of cell lysates were performed as described before [73]. In brief, cell lysate samples (20 µg per well) were resolved in 10% reducing SDS-PAGE with using NuPAGE™ 10%, Bis-Tris, Mini Protein Gel (Thermo Fisher Scientific, Waltham, MA, USA). Then the proteins were transferred from the gel onto the nitrocellulose membranes using the Semi-Dry Blotter V10-SDB (Biostep, Burkhardtsdorf, Germany). Membranes were incubated in the blocking buffer (5% non-fat dried milk, 0.05% Tween 20 in PBS) for 1h at room temperature, followed by incubation with mouse monoclonal anti-KAP1 (1:1000, clone 20C1, ab22553; Abcam, Cambridge, England) or mouse monoclonal anti-alpha-tubulin antibodies (1:1000, clone DM1A, T6199; Sigma-Aldrich, St. Louis, MO, USA) in PBS supplemented with 0.05% Tween 20 (PBS-T) for 1h at room temperature. After this, membranes were washed in PBS-T 2–3 times, and were incubated with secondary HRP-labeled anti-mouse antibodies (1:6000) (#12-349; Sigma-Aldrich, St. Louis, MO, USA). Membranes were rinsed four times in PBS-T, and the immunoreactive proteins were visualized with 1-Step Ultra TMB (3,3′,5,5′-tetramethylbenzidine)-Blotting Solution (Thermo Fisher Scientific, Waltham, MA, USA) according to the manufacturer’s instructions.

### 4.13. Statistical Analysis

GraphPad Prism 7.0 software was used for statistical analysis and graph creation. The applied statistical methods are described in the «Results» section. The value of *p* ≤ 0.05 was considered statistically significant. All experiments were repeated at least three times.

## Figures and Tables

**Table 1:** CD133 expression in cancer cell lines. The mean values of the percentages of CD133-positive cells (*n* = 5; mean ± SD).

Disease	Cell Line	CD133-Positive Population (%)
Non-small cell lung carcinoma	A549	0
	H23	0
	H358	0
	H460	0
	H1299	0
Colorectal carcinoma	Caco2	99.7 ± 0.3
	HT-29	69.9 ± 21.8
	SW480	0
	HCT116	0
	SW-837	0
Glioblastoma	LN-229	0
	T98G	0
	U-87 MG	0
Hepatocarcinoma	HUH7	93.4 ± 4.4
Kidney carcinoma	A704	0
Pancreas carcinoma	PANC-1	0
Thyroid carcinoma	FTC-133	5.7 ± 4.1
Breast carcinoma	MDA-MB-231	0
Neuroblastoma	IMR-32	0
Fibrosarcoma	HT-1080	0
Osteosarcoma	U-2 OS	0

**Table 2:** Molecular key regulators of CD133 revealed by in silico analysis. Final list of molecular key regulators (KRs), ranged by their total rank (maximal rank—12). Ranks of KRs were calculated by summing up the ranks of corresponding transcription factors (TFs) and master regulators (MRs).

Key Regulators	TFs Rank	MRs Rank	Total Rank
TRIM28	5	5	10
RELA	5	3	8
MYB	4	4	8
CREB1	4	2	6
REST	4	2	6
TP53	4	2	6
CEBPA	3	3	6
GABPB1	3	2	5
NANOG	2	2	4
E2F1	2	2	4
E2F3	2	2	4
E2F4	2	2	4
E2F7	2	2	4
EGR1	2	2	4
HIF1A	2	2	4
HMGA1	2	2	4

## Data Availability

The data presented in this study are available within the article text, figures and Supplementary Materials. Microarray data are available in the ArrayExpress database (http://www.ebi.ac.uk/arrayexpress, accessed on 19 May 2022) under the accession number E-MTAB-11779.

## Supplementary Materials

The supporting information can be downloaded at: www.mdpi.com/article/10.3390/ijms23179874/s1.
