# Supplementary material for: TRIM28 Is a Novel Regulator of CD133 Expression Associated with Cancer Stem Cell Phenotype
Source: Int J Mol Sci. 2022 Aug 30;23(17):9874. doi: 10.3390/ijms23179874 (PMC9456468; doi:10.3390/ijms23179874)
Supplement: Supplementary file 1 [file ijms-23-09874-s001.zip › ijms-1846784 supplementary, Proofread/Supplementary Figures S1-4.pdf]

SUPPLEMENTARY FIGURES S1-4

TRIM28 is a novel regulator of CD133 expression associated with cancer stem cell phenotype

Yan S. Kim[1], Daria M. Potashnikova[2], Alisa M. Gisina[1], Irina V. Kholodenko[1], Arthur T. Kopylov[3], Olga V. Tikhonova[3], Leonid K. Kurbatov[4], Aleena A. Saidova[2,5], Anna V. Tvorogova[6], Roman V. Kholodenko[7], Pavel V. Belousov[8,9], Ivan A. Vorobjev[6,10,11], Victor G. Zgoda[3], Konstantin N. Yarygin[1], Alexey Yu. Lupatov[1].

[1] Laboratory of Cell Biology, Orekhovich Institute of Biomedical Chemistry, Moscow, Russia.

[2] Cell biology and histology department, School of Biology, Lomonosov Moscow State University, Moscow, Russia

[3] Laboratory of Systems Biology, Orekhovich Institute of Biomedical Chemistry, Moscow, Russia.

[4] Transcriptome Analysis Group, Analytical Branch Department, Orekhovich Institute of Biomedical Chemistry, Moscow, Russia.

[5] Department of transcription factors, Engelhardt Institute of Molecular Biology of the Russian Academy of Sciences, Moscow, Russia.

[6] Laboratory of Cell Motility, Belozersky Research Institute of Physico-Chemical Biology, Lomonosov Moscow State University, Moscow, Russia.

[7] Laboratory of Molecular Immunology, Shemyakin - Ovchinnikov Institute of Bioorganic Chemistry of the Russian Academy of Sciences, Moscow, Russia.

[8] Endocrinology Research Centre, Moscow, Russia.

[9] Center for Precision Genome Editing and Genetic Technologies for Biomedicine, Engelhardt Institute of Molecular Biology of the Russian Academy of Sciences, Moscow, Russia

[10] Department of Biology, School of Sciences and Humanities, Nazarbayev University, Nur-Sultan, Kazakhstan.

[11] Laboratory of Biophotonics and Imaging, National Laboratory Astana, Nazarbayev University, Nur-Sultan, Kazakhstan.

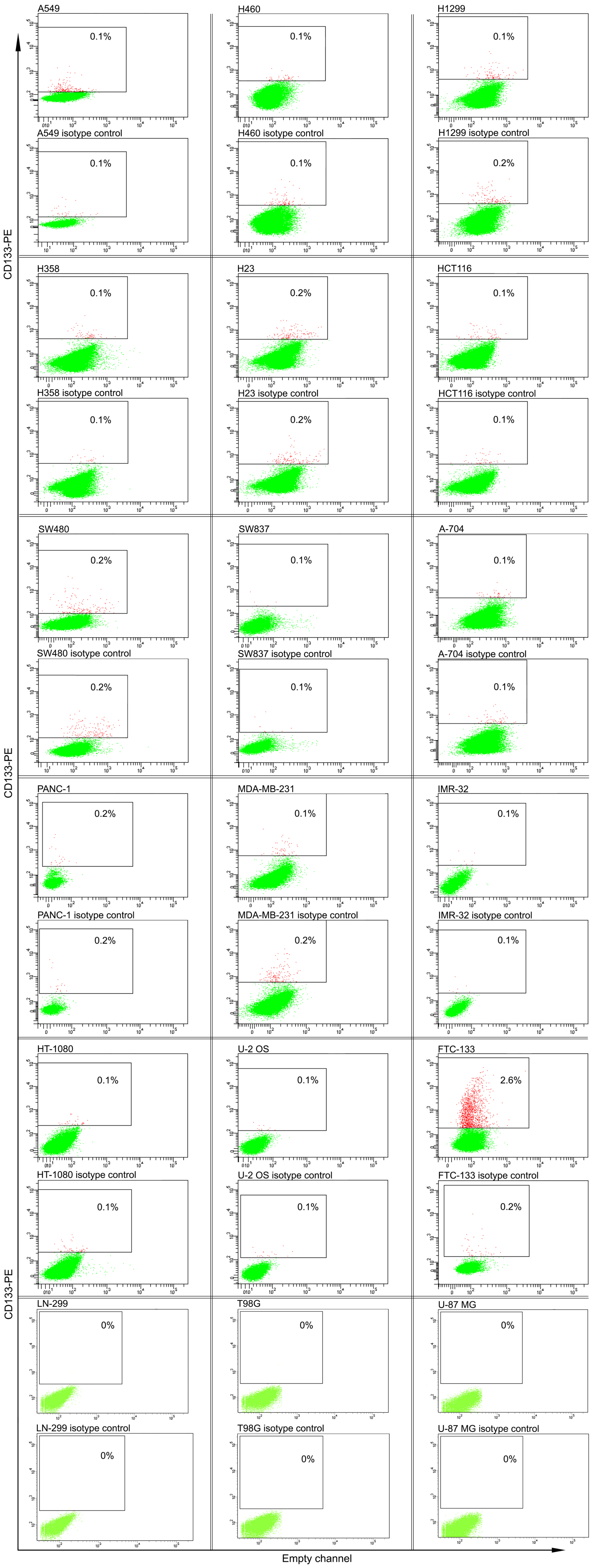

Supplementary figure S1. FACS analysis of CD133 expression in cancer cell lines. CD133 staining and the isotype control are presented for each cell line. Only cell lines including no or a minor CD133-positive population are shown.

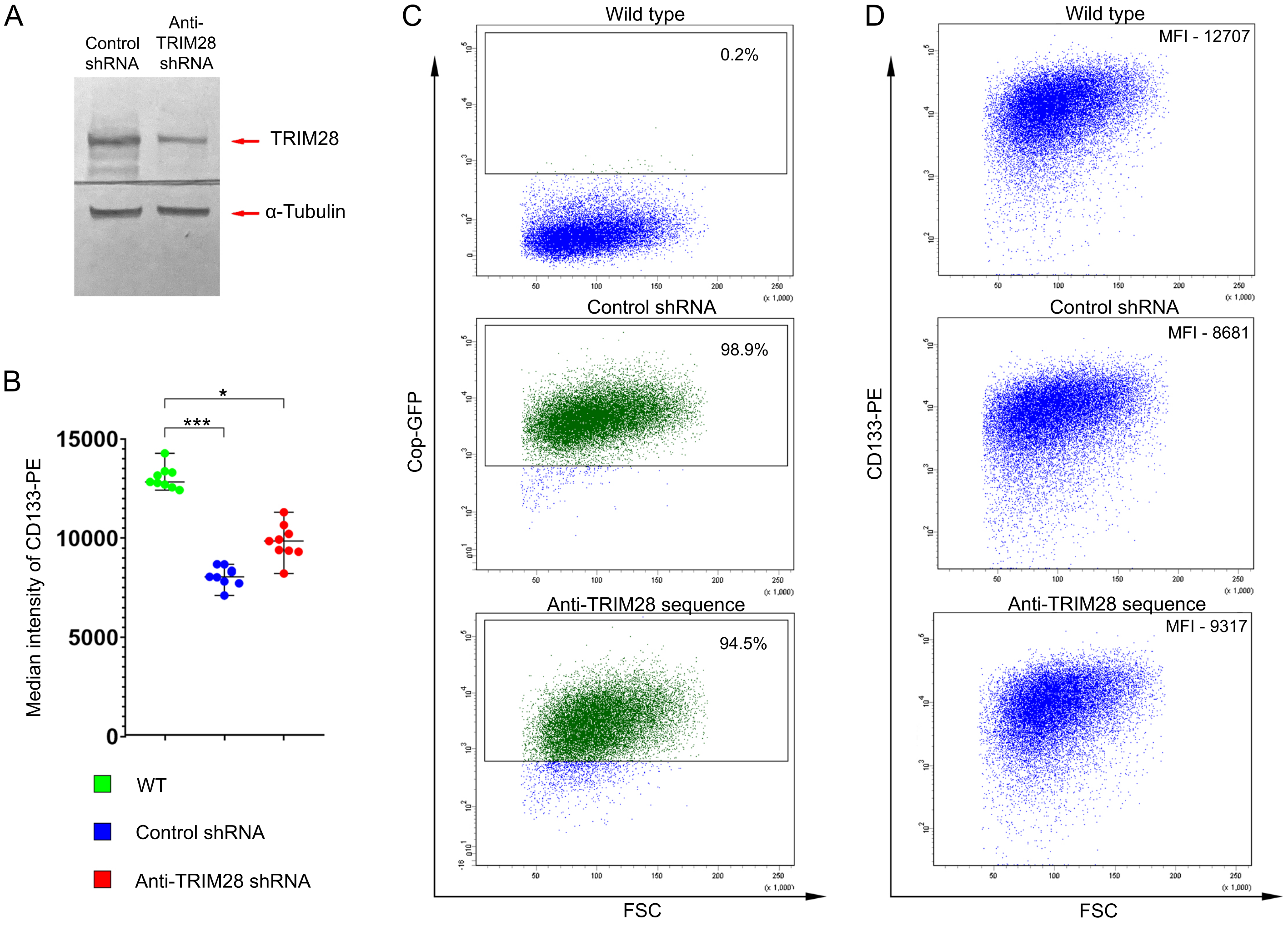

Supplementary figure S2. Downregulation of TRIM28 in Caco2 cells does not cause alteration of CD133 expression. (**A**) Verification of TRIM28 knockdown by Western blot. (**B**) CD133-PE median fluorescence intensity (MFI) measured in cells with TRIM28 knockdown and control groups (n=9 for each group, median and range). (**C**) FACS analysis of transduction efficiency using CopGFP reporter gene. (**D**) FACS analysis of CD133 expression in cells after TRIM28 downregulation. * $p \leq 0.05$; ** $p \leq 0.01$; *** $p \leq 0.001$.

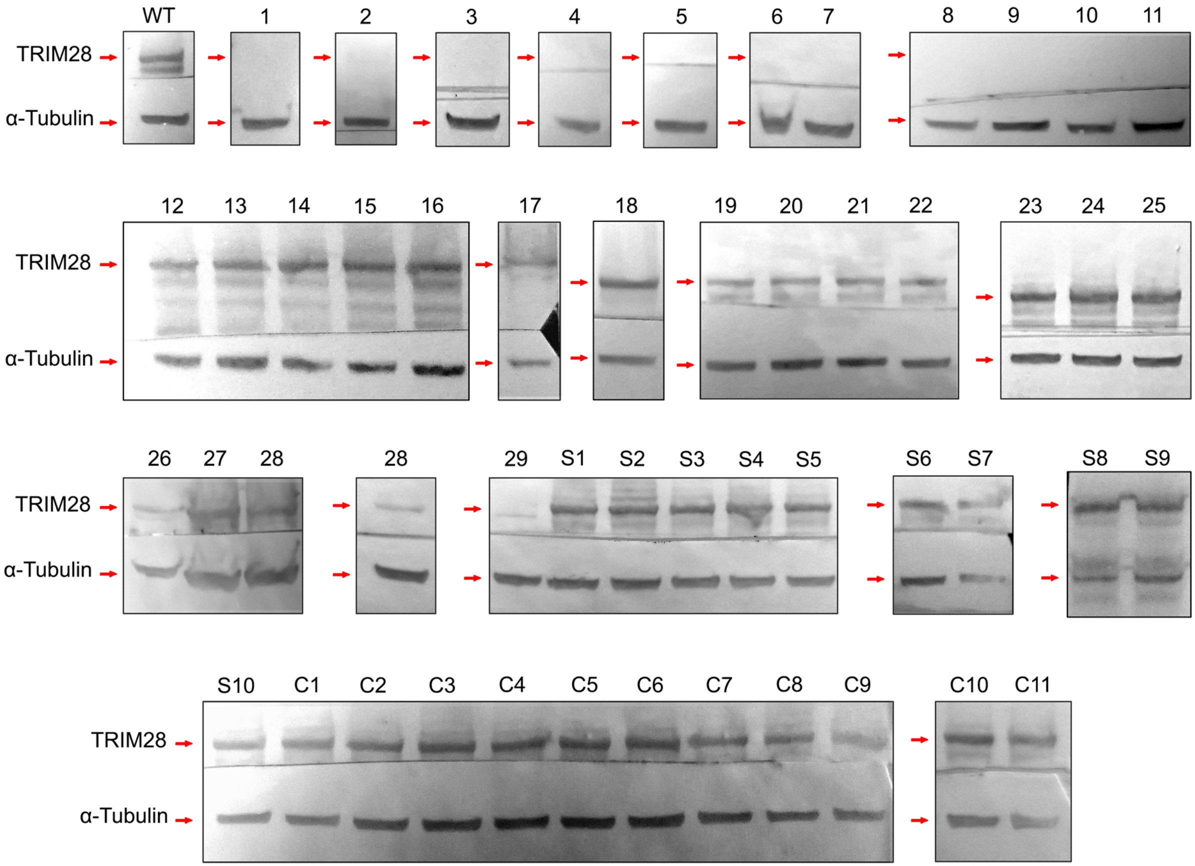

Supplementary figure S3. TRIM28 protein expression in Caco2 derived clones. *TRIM28*-knockout clones (No.1-11), clones with attempted but failed *TRIM28*-knockout (No.12-29), clones with attempted knockout using gRNA targeted to *tagRFP* non-mammalian gene (No.S1-S10), and clones generated without knockout procedure (No.C1-C11). Arrows indicate TRIM28 and alpha-tubulin bands. The figure composes nine Western blots. To prevent masking of tubulin band with lower TRIM28 band (caused apparently by partial degradation) all membranes were cut between alpha-tubulin and TRIM28 bands and processed with primary antibodies separately (with four exceptions – samples 1, 2, S8, S9).

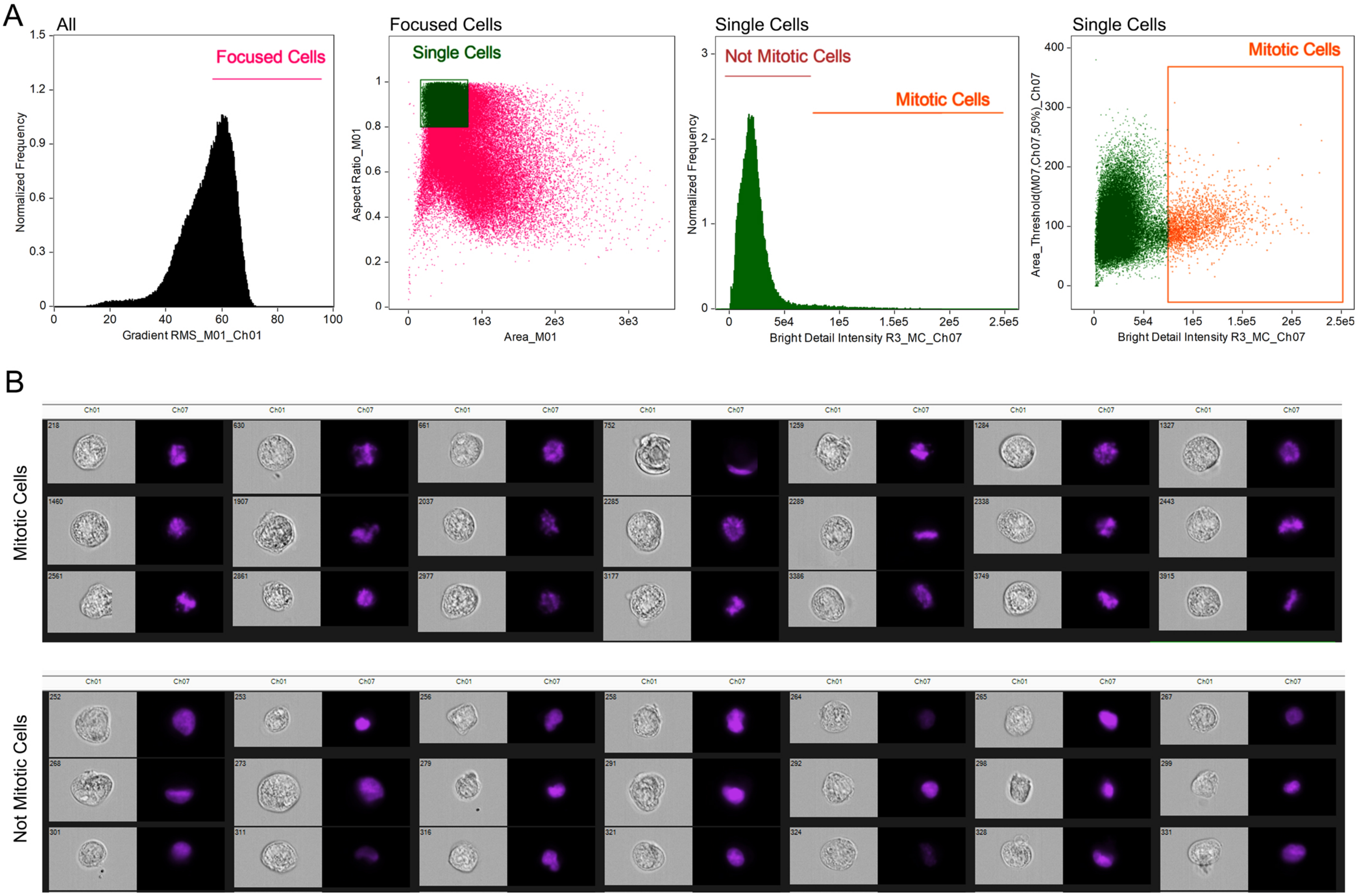

Supplementary figure S4. Adapted IDEAS Wizard «Cell Cycle - Mitosis» algorithm. (**A**) Focused cells were gated by «Gradient RMS» feature in the brightfield channel, followed by gating single cells on «Area»-«Aspect Ratio» in the same channel. Gating of mitotic cells was performed according to the IDEAS Wizard «Cell Cycle - Mitosis» algorithm with some changes. Since the used cancer cell lines are aneuploidy, we skipped the G2/M gating step and ignored «Area_Threshold», while identifying mitotic cells by «Bright Detail Intensity R3» feature only. We defined mitotic cells as those with the «Bright Detail Intensity R3» feature exceeding three standard deviation above the feature mean. (**B**) Brightfield and nuclear staining pictures of mitotic (top panel) and not mitotic cells (bottom panel). The top panel demonstrates a group of cells enriched with cells displaying condensed chromatin in various mitotic figures. The bottom panel demonstrates cells with no explicit mitotic figures.
