# Supplementary material for: TRIM28 Is a Novel Regulator of CD133 Expression Associated with Cancer Stem Cell Phenotype
Source: Int J Mol Sci. 2022 Aug 30;23(17):9874. doi: 10.3390/ijms23179874 (PMC9456468; doi:10.3390/ijms23179874)
Supplement: Supplementary file 1 [file ijms-23-09874-s001.zip › ijms-1846784 supplementary, Proofread/Supplementary Tables S1-3.pdf]

SUPPLEMENTARY TABLES S1-3

TRIM28 is a novel regulator of CD133 expression associated with cancer stem cell phenotype

Yan S. Kim[1], *Daria M. Potashnikova*[2], *Alisa M. Gisina*[1], *Irina V. Kholodenko*[1], *Arthur T. Kopylov*[3], *Olga V. Tikhonova*[3], *Leonid K. Kurbatov*[4], *Aleena A. Saidova*[2,5], *Anna V. Tvorogova*[6], *Roman V. Kholodenko*[7], *Pavel V. Belousov*[8,9], *Ivan A. Vorobjev*[6,10,11], *Victor G. Zgoda*[3], *Konstantin N. Yarygin*[1], *Alexey Yu. Lupatov*[1].

[1] Laboratory of Cell Biology, Orekhovich Institute of Biomedical Chemistry, Moscow, Russia.

[2] Cell biology and histology department, School of Biology, Lomonosov Moscow State University, Moscow, Russia

[3] Laboratory of Systems Biology, Orekhovich Institute of Biomedical Chemistry, Moscow, Russia.

[4] Transcriptome Analysis Group, Analytical Branch Department, Orekhovich Institute of Biomedical Chemistry, Moscow, Russia.

[5] Department of transcription factors, Engelhardt Institute of Molecular Biology of the Russian Academy of Sciences, Moscow, Russia.

[6] Laboratory of Cell Motility, Belozersky Research Institute of Physico-Chemical Biology, Lomonosov Moscow State University, Moscow, Russia.

[7] Laboratory of Molecular Immunology, Shemyakin - Ovchinnikov Institute of Bioorganic Chemistry of the Russian Academy of Sciences, Moscow, Russia.

[8] Endocrinology Research Centre, Moscow, Russia.

[9] Center for Precision Genome Editing and Genetic Technologies for Biomedicine, Engelhardt Institute of Molecular Biology of the Russian Academy of Sciences, Moscow, Russia

[10] Department of Biology, School of Sciences and Humanities, Nazarbayev University, Nur-Sultan, Kazakhstan.

[11] Laboratory of Biophotonics and Imaging, National Laboratory Astana, Nazarbayev University, Nur-Sultan, Kazakhstan.

Supplementary Table S1. Differentially expressed transcripts and proteins for CD133$^{+/high}$ and CD133$^{-/low}$ cell populations of Caco2, HT-29 and HUH7 cell lines. «Fold change» represents ratio of transcript or protein expression in CD133$^{+/high}$ to CD133$^{-/low}$ cells calculated from three biological repeats.

TRANSCRIPTS

HT-29

Gene name	*Fold change*	*Gene name*	*Fold change*
HLA-DMB	4.21	*CRYM*	2.12
CD133	2.97	*SLC4A4*	2.06
GPC4	2.95	*TOX2*	0.49
RGS2	2.25	*MAGEA2, MAGEA2B**	0.46
SNX10	2.17	*SCEL*	0.45

Caco2

Gene name	*Fold change*	*Gene name*	*Fold change*
AHSG	3.03	*AXIN2*	0.46
ALB	2.96	*GABARAPL1*	0.46
RBP4	2.70	*TACSTD2*	0.45
CYP4X1	2.62	*COL4A6*	0.45
CD133	2.57	*RGCC*	0.45
TFF3	2.32	*CEP55*	0.45
SCGN	2.31	*S100A14*	0.45
FRAS1	2.24	*PODXL*	0.44
TTR	2.17	*PRSS23*	0.44
BFSP1	2.12	*TAGLN*	0.44
TFF2	2.10	*LTB*	0.44
AFP	2.08	*FGF18*	0.43
SUSD3	2.04	*AC104698.1, LBH**	0.43
CHRDL2	2.01	*AL391319.1, SMOC2**	0.42
DEPP1	0.50	*FAM171B*	0.42
TPM1	0.50	*MYBPH*	0.42
COG3	0.50	*BASP1*	0.42
EDN3	0.50	*COTL1*	0.41
CCND2	0.50	*TFCP2L1*	0.40
PPIC	0.49	*CDC42EP3*	0.39
UTS2	0.49	*TUBB3, TUBB3P1**	0.38
ABAT	0.49	*MBNL3*	0.37
CAB39L	0.49	*NRP1*	0.36
C12orf75	0.49	*KCNJ16*	0.35
IGFL2	0.49	*ADAM8*	0.34
S100A6	0.48	*NPPB*	0.34
TNNC1	0.48	*BIK*	0.33
PTGS2	0.48	*CAMK1D*	0.30
NFE2	0.48	*OLR1*	0.30
PROCR	0.48	*MYL7*	0.27
TUBB2B	0.48	*SOCS2*	0.26
RHOU	0.47	*ACTG2*	0.26

DPYSL3	0.47	*BAMBI*	0.24
MYLPF	0.47	*ANXA1*	0.20
TMSB4XP4	0.46	*WNT6*	0.18
GPC1	0.46		

HUH7

Gene name	*Fold change*	*Gene name*	*Fold change*
CD133	5.58	*UNC5B*	2.26
BEX1	5.52	*PRSS23*	2.26
DMKN	4.82	*FERMT1*	2.21
BEX2	4.72	*TGFB2*	2.20
COL2A1	4.64	*DNALI1*	2.20
EPCAM	4.32	*TRPM6*	2.18
CRLF1	4.02	*CXCL1*	2.16
COTL1	3.98	*NEO1*	2.14
ERP27	3.66	*C3orf38*	2.11
PLBD1	3.25	*ICAM2*	2.11
NDRG4	3.24	*FOXO1*	2.08
TPD52L1	3.15	*LHX2*	2.08
GCA	3.13	*INCENP*	2.06
PRKCZ	3.12	*SULT1A2*	2.06
*AC093917.1, GBA3**	3.09	*HTRA3*	2.05
FSTL1	3.05	*CPE*	2.04
SLC1A3	3.03	*OGT*	2.03
TESC	3.00	*SLC7A7*	2.02
FST	2.96	*GPX8*	2.02
DKK1	2.89	*KITLG*	2.02
AUTS2	2.82	*SLC6A10P, SLC6A8**	0.49
*AC124309.1, MAGEL2**	2.78	*MBL2*	0.48
*KRT15, KRT19**	2.76	*C2, CFB**	0.47
SLIT2	2.74	*RTN4RL2*	0.47
MEST	2.67	*C3*	0.46
EMC10	2.64	*GREM2*	0.45
DUSP26	2.55	*FGL1*	0.40
MAGEA8	2.49	*NPTX2*	0.40
DPYSL5	2.38	*CFH, CFHR1**	0.38
ENPP4	2.38	*CSTA*	0.33
INPP5D	2.34	*NEURL3*	0.32
EDN1	2.34	*ARG1*	0.26
CAND2	2.33	*SERPINC1*	0.23

PROTEINS

HT-29

Gene name	*Fold change*	*Gene name*	*Fold change*
SEPT2	4.17	*PDAP1*	2.16
PSMD2	3.60	*TBCA*	2.10
ARPC5	3.50	*THRAP3*	2.06
EIF5A	2.89	*RPS17*	2.03

ALDH1A1	2.88	*HIBADH*	2.03
RPL31	2.84	*MTAP*	2.02
BAG2	2.77	*UBQLN1*	0.49
NDUFS3	2.51	*SORD*	0.49
RBBP7	2.45	*TMSB10*	0.48
ANP32E	2.33	*TMED9*	0.48
RBBP4	2.32	*SLC7A5*	0.47
DDAH1	2.31	*HNRNPAB*	0.46
PSMA4	2.29	*PODXL*	0.46
CAPRIN1	2.26	*IDH3A*	0.44
PSMD13	2.24	*SPINT2*	0.43
RUVBL2	2.23	*PDCD10*	0.40
NUDC	2.23	*GGCT*	0.40
TMSB4X	2.22		

Caco2

Gene name	*Fold change*	*Gene name*	*Fold change*
HMGB2	4.25	*RPL26*	0.47
RPS8	3.81	*ALDH2*	0.47
EIF4A3	3.53	*CNN2*	0.46
CLNS1A	2.73	*EIF3B*	0.46
SPEN	2.59	*LAP3*	0.46
RPS19	2.55	*SURF4*	0.45
ETFB	2.53	*RPN2*	0.45
FH	2.39	*TCOF1*	0.45
PPME1	2.36	*RAB7A*	0.44
GLS	2.28	*RAB11A*	0.44
PSME1	2.22	*DAD1*	0.44
RPS25	2.20	*AGR2*	0.42
PARK7	2.17	*LMNA*	0.41
MAP4	2.14	*PHB*	0.40
PRPF19	2.10	*ELAVL1*	0.40
PSMD3	2.04	*EIF3G*	0.38
NUMA1	2.02	*VCL*	0.34
CTTN	0.49	*TLN1*	0.31
EIF4G1	0.49	*DBI*	0.22
TAGLN	0.48		

HUH7

Gene name	*Fold change*	*Gene name*	*Fold change*
LRPPRC	10.26	*VIM*	2.26
AHNAK	5.51	*RAB7A*	2.25
RPS28	4.52	*TFG*	2.24
DDOST	4.19	*PSMC1*	2.23
IDH1	3.94	*SERPINH1*	2.21
PRKDC	3.78	*TLN1*	2.19
MYL12A	3.68	*SPTBN1*	2.18
YWHAG	3.60	*HSD17B4*	2.17
PHGDH	3.57	*FABP1*	2.17
HNRNPL	3.49	*ECHS1*	2.13

TOMM40	3.42	*USP5*	2.12
DBI	3.37	*VCL*	2.11
YWHAE	3.20	*PRDX4*	2.10
RPS3	3.15	*HSPA4*	2.09
TARDBP	3.04	*SRP14*	2.09
CKB	3.04	*RPN1*	2.07
S100A6	2.85	*S100P*	2.07
PRDX6	2.80	*EIF3A*	2.06
RPL17	2.77	*YWHAQ*	2.06
SEC61A1	2.75	*PGD*	2.02
UCHL1	2.72	*TMED9*	2.01
RPL10	2.71	*APEX1*	2.01
ALDH18A1	2.69	*TXNRD1*	2.01
GLUD1	2.65	*TMPO*	2.00
FABP5	2.65	*AK4*	0.50
SPTAN1	2.54	*ACAT2*	0.49
EEF1B2	2.47	*SSBP1*	0.48
HNRNPA3	2.45	*PCMT1*	0.48
SHMT2	2.45	*RPS19*	0.44
CCAR2	2.44	*STMN1*	0.42
CALR	2.42	*SF3A3*	0.42
ACTN4	2.39	*NME1-NME2**	0.38
TUFM	2.38	*RPL6*	0.37
GANAB	2.33	*HSPE1*	0.35
DPP3	2.31	*RPS17*	0.35
IMMT	2.31	*RPS14*	0.32
DDX39B	2.31	*TOMM22*	0.25
DNM1L	2.26		

* Transcripts or proteins could not be distinguished due to similarity of their sequences.

Supplementary Table S2. Final lists of transcription factors (TFs), master regulators (MRs), and molecular key regulators (KRs) arranged by rank.

Gene	TFs	MRs	Total rank	Gene	TFs	MRs	Total rank
Molecular key regulators				*Transcription factors (continued)*			
TRIM28	5	5	10	*BACH2*	2	0	2
RELA	5	3	8	*BCL6*	2	0	2
MYB	4	4	8	*BRCA1*	2	0	2
CREB1	4	2	6	*CDX2*	2	0	2
REST	4	2	6	*ESR1*	2	0	2
TP53	4	2	6	*FOXA2*	2	0	2
CEBPA	3	3	6	*FOXC1*	2	0	2
GABPB1	3	2	5	*HMGA2*	2	0	2
NANOG	2	2	4	*HNF4A*	2	0	2
E2F1	2	2	4	*HOXD12*	2	0	2
E2F3	2	2	4	*IKZF2*	2	0	2
E2F4	2	2	4	*IRF1*	2	0	2
E2F7	2	2	4	*LEF1*	2	0	2
EGR1	2	2	4	*MAF*	2	0	2
HIF1A	2	2	4	*MAFB*	2	0	2
HMGA1	2	2	4	*MAFG*	2	0	2
Transcription factors				*MAFK*	2	0	2
GCM2	6	0	6	*MEF2A*	2	0	2
IKZF1	5	0	5	*MEIS1*	2	0	2
KLF6	5	0	5	*NFE2*	2	0	2
BPTF	4	0	4	*NFE2L1*	2	0	2
CHURC1	4	0	4	*NFE2L2*	2	0	2
CTCF	4	0	4	*NFIA*	2	0	2
GLI1	4	0	4	*NFIC*	2	0	2
GLI2	4	0	4	*NR5A2*	2	0	2
GLI3	4	0	4	*NRF1*	2	0	2
GLIS1	4	0	4	*PATZ1*	2	0	2
GLIS2	4	0	4	*PDX1*	2	0	2
GLIS3	4	0	4	*POU2F1*	2	0	2
GTF2IRD1	4	0	4	*RBPJ*	2	0	2
HLTF	4	0	4	*RFX1*	2	0	2
KLF4	4	0	4	*SMAD4*	2	0	2
MAZ	4	0	4	*SRF*	2	0	2
MZF1	4	0	4	*TBX5*	2	0	2
PBX1	4	0	4	*TEAD1*	2	0	2
PBX2	4	0	4	*TFDP1*	2	0	2
PBX3	4	0	4	*ZBTB16*	2	0	2
RREB1	4	0	4	*ZBTB33*	2	0	2
SOX2	4	0	4	*Master regulators*			
SRY	4	0	4	*ADAM10*	0	3	3
TCF3	4	0	4	*CAPNS1*	0	3	3
TFAP2A	4	0	4	*CDK2*	0	3	3
ZIC1	4	0	4	*MAPK3*	0	3	3
ZIC2	4	0	4	*PTGES*	0	3	3
ZIC3	4	0	4	*ADAM17*	0	2	2

ZNF263	4	0	4	***CAMK2A***	0	2	2
DBP	3	0	3	***CAMK2B***	0	2	2
ELF1	3	0	3	***CAMK2D***	0	2	2
ELF2	3	0	3	***CAMK2G***	0	2	2
ELF4	3	0	3	***CHUK***	0	2	2
ELK1	3	0	3	***CSK***	0	2	2
ELK3	3	0	3	***DRD1***	0	2	2
ELK4	3	0	3	***DVL2***	0	2	2
ERG	3	0	3	***EGF***	0	2	2
ETS1	3	0	3	***EGFR***	0	2	2
ETS2	3	0	3	***ERBB2***	0	2	2
ETV4	3	0	3	***FRAT1***	0	2	2
ETV6	3	0	3	***GRIN1***	0	2	2
ETV7	3	0	3	***GSK3B***	0	2	2
FLI1	3	0	3	***IKBKB***	0	2	2
GABPA	3	0	3	***IKBKG***	0	2	2
GFI1	3	0	3	***ITGA2B***	0	2	2
HDX	3	0	3	***ITGB3***	0	2	2
HES1	3	0	3	***MAPK1***	0	2	2
HOMEZ	3	0	3	***MAPK11***	0	2	2
HOXB13	3	0	3	***MAPK12***	0	2	2
HOXC13	3	0	3	***MAPK13***	0	2	2
HSF1	3	0	3	***MLST8***	0	2	2
NR5A1	3	0	3	***MTOR***	0	2	2
POU1F1	3	0	3	***NEK7***	0	2	2
POU6F1	3	0	3	***PASK***	0	2	2
SIX1	3	0	3	***PKN2***	0	2	2
SP1	3	0	3	***PTEN***	0	2	2
SP3	3	0	3	***PTK2***	0	2	2
SP4	3	0	3	***PTPN12***	0	2	2
SPI1	3	0	3	***RANBP2***	0	2	2
SPIB	3	0	3	***RPS6KB1***	0	2	2
TFCP2	3	0	3	***RPTOR***	0	2	2
AIRE	2	0	2	***SENP1***	0	2	2
BACH1	2	0	2	***TNFRSF1A***	0	2	2

Supplementary Table S3. Sequences for lentivirus knockdown and CRISPR/CAS9-mediated knockout (*TRIM28* NCBI Reference Sequence: NG_046945.1). (**A**) Anti-*TRIM28* shRNA sequences for lentivirus knockdown and negative control sequences against firefly luciferase (Luc). Target sequences are highlighted with yellow. (**B**) Anti-*TRIM28* sgRNA sequences for CRISPR/CAS9-mediated knockout and negative control sequences against *tagRFP*. Target sequences are highlighted with yellow.

A

Gene		shRNA sequence (5′-3′)
TRIM28	sense	GATCCGCATGAACCCCTTGTGCTGTTCAAGAGACAGCACAAGGGGTTCATGCTTTTTG
	antisense	AATTCAAAAAGCATGAACCCCTTGTGCTGTCTCTTGAACAGCACAAGGGGTTCATGCG
Luc	sense	GATCCCGTACGCGGAATACTTCGATTCAAGAGATCGAAGTATTCCGCGTACGTTTTTG
	antisense	AATTCAAAAACGTACGCGGAATACTTCGATCTCTTGAATCGAAGTATTCCGCGTACGG

B

Gene		sgRNA sequence (5′-3′)
TRIM28 gRNA_1	sense	caccGTTTGCACTCGGCCTGTAG
	antisense	aaacCTACAGGCCGAGTGCAAAC
TRIM28 gRNA_2	sense	aaacCTGGCGGCGAAAAGCGCTCC
	antisense	caccGGAGCGCTTTTCGCCGCCAG
TRIM28 gRNA_3	sense	caccGACCGGTAAGTACGAAGTGAT
	antisense	aaacATCACTTCGTACTTACCGGTC
tagRFP	sense	caccGTCACCACATACGAAGACGG
	antisense	aaacCCGTCTTCGTATGTGGTGAC
